# *Hordeum chilense* genome, a useful tool to investigate the endosperm yellow pigment content in the *Triticeae*

**DOI:** 10.1186/1471-2229-12-200

**Published:** 2012-11-02

**Authors:** Cristina Rodríguez-Suárez, Sergio G Atienza

**Affiliations:** 1 Instituto de Agricultura Sostenible, IAS-CSIC, Apdo 4084, Córdoba, E-14080, Spain

**Keywords:** Yellow pigment content (YPC), Macrocolinearity, Candidate genes, *H*. *chilense*, *H*. *vulgare*

## Abstract

**Background:**

The wild barley *Hordeum chilense* fulfills some requirements for being a useful tool to investigate the endosperm yellow pigment content (YPC) in the *Triticeae* including its diploid constitution, the availability of genetic resources (addition and deletion stocks and a high density genetic map) and, especially, its high seed YPC not silenced in tritordeums (amphiploids derived from *H*. *chilense* and wheat). Thus, the aim of this work was to test the utility of the *H*. *chilense* genome for investigating the YPC in the *Triticeae*.

**Results:**

Twelve genes related to endosperm carotenoid content and/or YPC in grasses (*Dxr*, *Hdr* [synonym *ispH*], *Ggpps1*, *Psy2*, *Psy3*, *Pds*, *Zds*, *e*-*Lcy*, *b*-*Lcy*, *Hyd3*, *Ccd1* and *Ppo1*) were identified, and mapped in *H*. *chilense* using rice genes to identify orthologs from barley, wheat, sorghum and maize. Macrocolinearity studies revealed that gene positions were in agreement in *H*. *vulgare* and *H*. *chilense*. Additionally, three main regions associated with YPC were identified in chromosomes 2H^ch^, 3H^ch^ and 7H^ch^ in *H*. *chilense*, the former being the most significant one.

**Conclusions:**

The results obtained are consistent with previous findings in wheat and suggest that *Ggpps1*, *Zds* and *Hyd3* on chromosome 2H^ch^ may be considered candidate genes in wheat for further studies in YPC improvement. Considering the syntenic location of carotenoid genes in *H*. *chilense*, we have concluded that the H^ch^ genome may constitute a valuable tool for YPC studies in the *Triticeae*.

## Background

*Hordeum chilense* Roem. et Schultz. is a diploid wild barley included in the section Anisolepis Nevski, native to Chile and Argentina. This species has been used as a new source of cytoplasmic male sterility for hybrid wheat production [1-3] and it shows other potentially useful traits for wheat breeding, including immunity to *Septoria tritici* blotch and high seed yellow pigment content (YPC) [4-6]. The compatibility of the *H*. *chilense* genome with *Triticum* spp. is high, and fertile amphiploids, named tritordeums (×Tritordeum Ascherson et Graebner), have been developed from the hybrid between *H*. *chilense* and durum or common wheat [7,8]. Tritordeums are subjected to a breeding program at IAS-CSIC conducted by Prof. A. Martín with the goal of obtaining a new crop. Additionally, they are also useful as a genetic bridge to transfer *H*. *chilense* genes to both common and durum wheat [9].

One of the most valuable traits of this new cereal is the high endosperm YPC given by *H*. *chilense* genes. The YPC, mainly caused by carotenoids, is also very important in durum wheat due to its relation to semolina quality [10-13]. In addition, the consumption of carotenoid-rich foods has been associated with a reduced risk of developing certain types of diseases [14,15]. Besides, carotenoids with unsubstituted β-ionone rings, such as β-carotene, have provitamin A activity [16] while other carotenoids such as lutein may prevent age-related macular degradation [17,18]. Accordingly, seed carotenoid content is receiving more attention with the goal of developing new functional foods.

The carotenoid biosynthesis pathway is well known in plants [16,19-22]. Candidate gene studies in maize have shown the role of several carotenoid-related genes in endosperm carotenoid content using quantitative trait locus (QTL) and association studies. Indeed, several genes including *Psy1*[23-25]; *Zds*[25]; *eLcy*[26]; *Ziso*[27] and *Hyd3*[28] have been related to endosperm carotenoid content. Genes from both upstream and downstream pathways have also been investigated at the transcriptomic level in maize [29].

Despite the importance of YPC in durum wheat and tritordeums and the positive correlation between YPC and carotenoid content, candidate gene studies for YPC in both species are mostly based on *Phytoene synthase 1* gene (*Psy1*). PSY1 catalyzes the first step of the carotenoid biosynthetic pathway and it is considered a limiting factor for carotenoid production [18,30]. Since the first reports about the location of *Psy1* to chromosomes 7A and 7B in wheat [31,32], a considerable amount of work has demonstrated the role of this gene in durum wheat YPC [33-39]. In addition, the lycopene *epsilon cyclase* gene (*eLcy*) has been co-localized with a QTL for YPC in wheat [35]. Nevertheless the role of other candidate genes for YPC in durum wheat remains largely unexplored. Given the high level of macrocolinearity among grasses, the knowledge gained in any species may be useful in others. This is more evident within the *Triticeae* due to the close relationship among these species. Indeed, *Triticeae* species are represented as a single genome in comparative studies [40,41].

In this context *H*. *chilense* may be a useful species to investigate the role of carotenoid-related genes in the YPC in the *Triticeae*. First, it exhibits a high YPC which is not silenced in wheat background, as evidenced in tritordeums [10,42,43]. Second, the diploid constitution of this species and the existence of *H*. *chilense*-wheat chromosome addition lines [44] and other deletion stocks [45,46] makes the identification of candidate genes more accessible. Third, a high density genetic map has been recently developed using *H*. *chilense*-derived DArT markers [47] which allows efficient candidate gene mapping and comparative studies.

The objective of this work is to determine whether *H*. *chilense* could be useful for investigating the endosperm YPC in the *Triticeae*. For this purpose we aimed to identify and locate a set of carotenoid-related genes previously studied in other grasses and to determine the macrocolinearity between *H*. *chilense* and barley for these genes. In the second place, we aimed to identify the main regions for YPC in *H*. *chilense* and to discuss the results obtained in relation to wheat.

## Methods

### Plant material

*Hordeum chilense* accession lines H1 and H7 from the collection of the Instituto de Agricultura Sostenible (IAS-CSIC, Córdoba) were used for polymorphism detection in the selected genes. DNA of 92 F_7_-Recombinant Inbred Lines (RILs) derived from the cross H1 × H7 was used for genetic mapping of the gene-based markers designed. These lines were sown in small pods with five seeds each. After initial growing, RILs were transplanted to field conditions using a randomized plot design with two replications.

For physical mapping, the following common wheat cv. Chinese Spring (CS)-*H*. *chilense* addition lines were used: with complete chromosomes 1H^ch^, 4H^ch^, 5H^ch^, 6H^ch^ or 7H^ch^ (named CS MA 1H^ch^-1 H^ch^S, CS DA4H^ch^, CS DA5H^ch^, CS DA6H^ch^ and CS DA7H^ch^, respectively, where MA refers to monosomic addition and DA to disomic addition), and (CS)-*H*. *chilense* ditelosomic addition lines CS DA1H^ch^S, CS DA2H^ch^S, CS DA5H^ch^L, CS DA6H^ch^S, CS DA7H^ch^α, CS DA7H^ch^β.

Leaf tissue was harvested at tillering stage, frozen in liquid nitrogen, and stored at -80°C. Genomic DNA was extracted using the CTAB method according to [48].

### Primer design

The twelve genes were selected from the main steps of the carotenoid biosynthetic pathway but also from upstream and downstream pathways, considering previous results obtained in other grasses, mainly maize. Rice genes were used as a query to identify orthologous genes in maize, sorghum, wheat and/or barley (see Additional file 1). Nucleotide sequences were aligned to design primers in the conserved regions of each gene. *Psy1* sequence of *H*. *chilense* was available from previous works [31,49]. Sequences were aligned using Edialign program ( http://emboss.sourceforge.net/index.html) and edited using GeneDoc software ( http://www.psc.edu/biomed/genedoc). Sequence identity searches were performed at the NCBI ( http://www.ncbi.nlm.nih.gov) using BLAST. Primer pairs were designed using Primer3plus software [50] on exonic regions flanking at least one intron when possible.

### PCR amplification and polymorphism detection

PCR amplifications were performed in 25 μl reactions consisting of 0.625 units of DNA polymerase (Biotools B&M Labs, Madrid, Spain), 1× PCR buffer, 1.6 mM MgCl_2_, 320 mM dNTPs (Promega, Madison, WI, USA), 0.6 mM of each primer and 50 ng of genomic DNA. PCR were carried out as follows: 5 min at 94°C, 35 cycles of 30 s at 94°C, 30 s at 58°C (54°C for *Zds*) and 1 min at 72°C followed by 7 min at 72°C. For allele-specific amplifications with tetra-primer PCR, the number of cycles was increased to 40 and the annealing temperature was set at 60°C. Amplification and digestion of *Psy1* gene were carried out as described by [31]. PCR products were resolved on 2% agarose gels, stained with ethidium bromide and visualized under UV light. All primers used in this work are summarized in Table 1.

**Table 1:** Primers designed for amplification and mapping of the candidate genes

**Primer**	**Sequence 5’**-**3’**	**Primer**	**Sequence 5’**-**3’**
GGPPS1-F1	GCCAGCGTCGACTCCTAC	PSY3-F	TCGACGAGCTGTACCTCTACTG
GGPPS1-R1	GGAACAGCAACCCAATTGAT	PSY3-R	CTCGATCTCGTCCAGGATCT
GGPPS1-spF	CGTATGCCTTTCTAAGAAGTG	HYD3-F3	CCTCCGTGTACTACCGCTTC
GGPPS1-spR	GGAGATACCTATGCAAATCAT	HYD3-R2	CGAACTTGTCCATGTGGTGT
DXR-F1	GAGCATGGGGAAGAAAATCA	ZDS-F2	TGTCCCAGGGATCAAAAGAC
DXR-R1	GGTGACCTCGGAGCAATAGA	ZDS-R2	AGCTTGGATCAGGGAACCTT
HDR-F2	GGCATTGCAAATCAAACAAC	LBC-F2	CAAGCTCAAGTCCACCATGA
HDR-R2	TTTCCTGGTCCAATCCTTTG	LBC-R2	TGTCTAGAAACCGCACGATG
LEC-F2	CTGGACAATATTTGCCTGGAA	LBC-spF	GCGACTCCCACCTCCCT
LEC-R2	GGAGGTGTCTGACGAGGTTC	LBC-spR	GATCGCGGACCCCTCG
PDS-F1	TACAGGTCGTGATTGCTGGT	CCD-F1	TTGATCCTACAAAGAAAGCTCGT
PDS-R1	GGGAAATCAAAACGGCTGTA	CCD-R1	CAGCTCATTCCCGAAGTTCT
PSY2-F	ATTGCTCCGGACTCAAAGG	CCD-spR	GAAGTTCTCCAGCTTGTCGG
PSY2-R	TTGTAGTCGTTCGCCTCGAT	PPO1-F1	AGCTTCGAGCAGCAGTGG
PSY2-spR	GAGTCAACAATGCTTGAATGA	PPO1-R1	GTGGTGCGCGAAGAAGAT

Nucleotide sequences were obtained by direct sequencing or by cloning if more than one product was observed in the amplifications. For cloning, pGEMT-Easy vector (Promega, Madison, WI) was used to transform competent *Escherichia coli* (DH5α) cells. Plasmids were isolated and purified using Illustra plasmid Prep Mini Spin Kit (GE Healthcare, UK Ltd, UK).

Digestion reactions with restriction enzymes *HhaI*, *HinfI*, *MboII*, *NheI* and *TasI* (Takara Bio Inc., Japan) were carried out following manufacturer’s instructions using 10 μl of PCR in a final volume of 20 μl.

### Identification of genomic regions for endosperm yellow pigment content (YPC)

YPC was determined according to the AACC method 14–50 [51] in 71 F_7_-RILs, which yielded a sufficient amount of seed. Briefly, approximately 150 mg of flour was used to evaluate YPC. Pigment extraction was performed using water-saturated n-butanol in a 1:10 (w/v) ratio. Samples were centrifuged at 14,000 g for 10 min and the pigment was measured by absorbance reading at 435.8 nm. All determinations were performed in duplicate.

A DArT-based *H*. *chilense* map [47] constituted the basis for mapping. A framework map composed by markers spaced 3–5 cM was considered to determine trait-marker associations and QTL mapping since the use of very proximal markers results in highly similar LOD scores while it signifies a huge increase in the number of calculations. Gene-based markers were integrated in the *H*. *chilense* map using JoinMap 4.0 mapping software [52]. After linkage analysis, the position of each candidate gene within a chromosome was determined using the fixed order option, the Monte Carlo Maximum Likelihood Mapping algorithm (ML-mapping) module and the Kosambi mapping function.

Associations between markers and YPC were determined using the Kruskal-Wallis (KW) test and MapQTL 4.0 [53]. Regions showing at least five markers associated with YPC at *p*<0.01 were considered as YPC regions but further analyses were performed to identify the most significant ones. Marker cofactors were automatically selected using the automatic cofactor selection (ACS) tool at *p*<0.05. For both Interval Mapping (IM) and Multiple QTL Mapping (MQM), the genome-wide significance threshold was estimated using the permutation test with the following parameters: the walk speed was set to 3 cM; the number of permutations was set to 500 and the significance level to 0.05. The QTL position was estimated at the peak LOD with a 2-LOD support interval [54]. The map figure included in the manuscript was produced using MapChart software [55].

## Results

### Gene-based marker design, amplification and mapping

A total of twelve genes not previously mapped in *H*. *chilense* were selected for this work: 1-deoxy-D-xylulose 5-phosphate reductoisomerase (*Dxr*) and 4-hydroxy-3-methylbut-2-enyl diphosphate reductase (*Hdr* [synonym *ispH*]) from the MEP pathway; geranyl geranyl pyrophosphate synthase (*Ggpps1*) for geranylgeranyl diphosphate synthesis; phytoene synthase 2 (*Psy2*), phytoene synthase 3 (*Psy3*), phytoene dehydrogenase (*Pds*), *zeta*-carotene desaturase (*Zds*), lycopene epsilon cyclase (*e*-*Lcy*), lycopene *beta* cyclase (*b*-*Lcy*) and *beta*-carotene hydroxylase 3 (*Hyd3*) from the carotenoid biosynthetic pathway; the carotenoid cleavage dioxygenase 1 gene (*Ccd1*) mediating the degradation of carotenoids to apocarotenoids and polyphenol oxidase 1 gene (*Ppo1*) implicated in plant tissue enzymatic browning.

Rice genes ( http://www.gramene.org) were used as a query to identify orthologs from barley, wheat, sorghum and maize. Exon-intron boundaries and highly conserved regions were recognized by the alignment of each sequence set. Primers were designed on conserved exonic regions to amplify at least one intron and maximize polymorphism detection. Amplifications with the primers designed (Table 1) were carried out using the parental lines H1 and H7 as template. PCR products were sequenced to confirm their identity and examined for polymorphisms by comparing both parental lines.

All the candidate genes were successfully amplified and sequenced in *H*. *chilense* [GenBank: JQ922078-JQ922105]. Differences between H1 and H7 sequences were found for all the candidate genes, these being in most cases Single Nucleotide Polymorphisms (SNP) (Table 2). These SNPs were used to develop either Cleaved Amplified Polymorphism (CAP) markers for *Zds*, *Hyd3*, *Pds*, *Hdr*, *Psy3* and *e*-*Lcy* or to design allele-specific primers. Specific amplifications were obtained by using tetra-primer PCR (to amplify both H1 and H7 alleles) for *Ggpps1* (GGPPS1-F1/ GGPPS1-R1/ GGPPS1-spF/ GGPPS1-spR) and *b*-*Lcy* (LBC-F2/ LBC-R2/ LBC-spF/ LBC-spR). Alternatively, specific reverse primers CCD-spR and PSY2-spR were designed to use in combination with CCD-F1 and PSY2-F, respectively, to obtain PCR amplification exclusively in one of the parental alleles. Finally, length polymorphisms were detected for *Dxr* (14 bp insertion in H7 sequence compared to H1) and *Ppo1*. Regarding *Ppo1*, two sequences were amplified in H1 with primer pair PPO1-F1/PPO1-R1 differing in two SNPs (JQ922103 and JQ922104), both of them having in common a deletion of 23 bp compared to H7 (JQ922105). This was the length polymorphism scored in the mapping population.

**Table 2:** Polymorphism between H1 and H7 lines used for mapping

**Gene**	**Accession numbers**		**Polymorphism detection**	
	**H1**	**H7**	**Genetic mapping**	**Physical mapping**
*Dxr*	JQ922078	JQ922079	Allele size	-
*Hdr*	JQ922096	JQ922097	MboII digestion	MboII digestion
*Ggpps1*	JQ922098	JQ922099	Allele specific primers	Allele specific primers
*Psy2*	JQ922084	JQ922085	Allele specific primers	Allele specific primers
*Psy3*	JQ922082	JQ922083	HhaI digestion	-
*Pds*	JQ922092	JQ922093	HhaI digestion	-
*Zds*	JQ922090	JQ922091	HinfI digestion	TasI digestion
*e*-*Lcy*	JQ922094	JQ922095	HhaI digestion	
*b*-*Lcy*	JQ922088	JQ922089	Allele specific primers	-
*Hyd3*	JQ922080	JQ922081	NheI digestion	-
*Ccd1*	JQ922086	JQ922087	Allele specific primers	Allele specific primers
*Ppo1*	JQ922103	JQ922105	Allele size	-
	JQ922104			

Polymorphism between H1 and H7 lines of *H*. *chilense* used for genetic and physical mapping of the candidate genes selected in this work. GeneBank accession number for each sequence is given. *Psy1* is not included as sequencing and mapping have been reported elsewhere.

**Figure 1:**
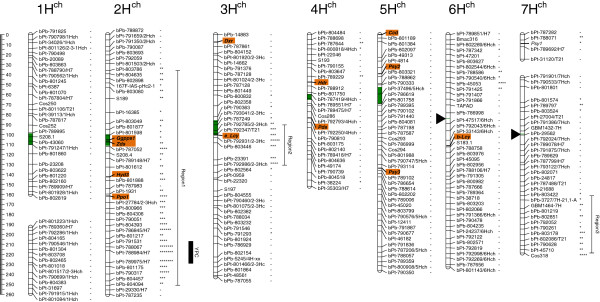
**MarkerYPC association in the KruskalWallis** (**KW**) **analysis.** To determine marker-YPC associations, a framework map composed by markers spaced around 3–5 cM was considered from the map developed by [47]. The estimated centromere position is shown as a coloured segment within each chromosome. In chromosomes 6H^ch^ and 7H^ch^ centromeres were located in a single position since markers belonging to different chromosome arms mapped at the same position. For chromosomes 6H^ch^ and 7H^ch^, the estimated centromere locations are shown as black triangles. The position of the candidate genes mapped in this work is highlighted using a coloured box. The significance of the KW analysis is shown: - Not significant; *0.1; **0.05; ***0.01; ****0.005; *****0.001; ******0.0005; *******0.0001. Three locations with at least five markers associated with YPC were considered as YPC regions (Regions 1 to 3). The most significant zone within these regions was determined using MQM analysis and permutation test (identified as YPC at 2H^ch^L) and its position estimated at the peak LOD with a 2-LOD support interval.

All gene-based markers were located in the *H*. *chilense* map (Figure 1) in the following chromosomes: 2H^ch^ (*Ggpps1*, *Zds*, *Hyd3* and *Ppo1*); 3H^ch^ (*Dxr* and *eLcy*); 4H^ch^ (*Hdr* and *Pds*); 5H^ch^ (*Ccd1*, *Psy2* and *Psy3*); 6H^ch^ (*bLcy*). The position (cM) of each candidate gene in the map is shown in Table 3. Both *Ggpps1* and *Zds* were tightly linked and mapped near the centromere which was estimated in a 11 cM window [47] while *Hyd3* and *Ppo1* were mapped to the long arm of chromosome 2H^ch^ at 142.38 cM and 161.56 cM respectively. The *eLcy* gene was located in the vicinity of the chromosome 3H^ch^ centromere while *Dxr* was mapped to the distal part of the 3H^ch^S arm (Figure 1). *Hdr* and *Pds* genes were mapped to 4H^ch^S and 4H^ch^L arms respectively. *Ccd1* (distal) and *Psy2* (39.06 cM) were mapped to chromosome 5H^ch^S while *Psy3* was mapped to the long arm of this chromosome (133.75 cM). Finally, *bLcy* was mapped to the long arm of chromosome 6H^ch^ (111.2 cM) since the centromere is estimated at 84.0 cM [47]. Also, *Psy1* is located on chromosome arm 7H^ch^α as shown in previous studies using physical mapping [31,47].

**Table 3:** **Macrocolinearity between *H***. ***chilense *and *H***. ***vulgare *for the candidate genes selected**

**Gene**	**Identifier**^**1**^	**Rice LOC**^**2**^	**Rice RAP**^**3**^	***Sorghum***^**3**^	***Brachypodium***^**3**^	***H. vulgare***^***4***^		***H. chilense***^***5***^	
						**Chr**	**cM**	**Chr**	**cM**
*Ggpps1*	POG_ORTHOMCL5142	LOC_Os07g39270	**Os07g0580900**	**Sb02g037510**	**Bradi1g23510**	2	59.21	2H^ch^C	105.35
*Zds*	POG_ORTHOMCL7646	LOC_Os07g10490	Os07g0204900	Sb02g006100	Bradi1g54390	*nd*	*nd*	2H^ch^C	107.36
*Hyd3*	POG_ORTHOMCL15690	LOC_Os04g48880	Os04g0578400	Sb06g026190	**Bradi5g19130**	2	90.1	2H^ch^L	142.38
*Ppo1*	POG_ORTHOMCL2213	LOC_Os04g53300	Os04g0624650	**Sb06g025570**/ **Sb06g025580**	**Bradi5g22300**	2	117.9	2H^ch^L	161.56
*e*-*Lcy*	POG_ORTHOMCL17555	LOC_Os01g39960	**Os01g0581300**	**Sb03g026020**	**Bradi2g41890**	3	55.57	3H^ch^C-L	100.47
*Dxr*	POG_ORTHOMCL8746	LOC_Os01g01710	**Os01g0106900**	**Sb03g008650**	**Bradi2g00650**	3	0	3H^ch^S	7.45
*Pds*	POG_ORTHOMCL8451	LOC_Os03g08570	**Os03g0184000**	Sb06g030030	**Bradi1g72400**	4	89.39	4H^ch^L	83.39
*Hdr*	POG_ORTHOMCL5875	LOC_Os03g52170	Os03g0731850	**Sb01g009140**	**Bradi1g09710**	4	38.63	4H^ch^S	47.49
*Psy3*	POG_ORTHOMCL18373	LOC_Os09g38320	**Os09g0555500**	**Sb02g032370**	**Bradi4g37520**	5	122.38	5H^ch^L	133.75
*Ccd1*	POG_ORTHOMCL13689	LOC_Os12g44310	Os012g0640600	**Sb01g047540**	**Bradi4g00330**	5	0.0	5H^ch^S	0.0
*Psy2*	POG_ORTHOMCL13713	LOC_Os12g43130	**Os12g0626400**	**Sb09g022310**	**Bradi4g01100**	5	26.28	5H^ch^S	39.06
*b*-*Lcy*	POG_ORTHOMCL11455	LOC_Os02g09750	**Os02g0190600**	**Sb04g006120**	Bradi3g06600	6	54.6	6H^ch^L	111.21
*Psy1*	POG_ORTHOMCL6706	LOC_Os06g51290	**Os06g0729000**	**Sb10g031020**	**Bradi1g29590**	7	166.56	7H^ch^α	8.93

(^1^) *Poaceae* orthologous group corresponding to each candidate gene; (^2^) MSU rice database identifiers of rice genes used as query to identify the *Poaceae* orthologous group; (^3^) Rice, *Sorghum* and *Brachypodium* genes included in each *Poaceae* orthologous group. For nomenclature of rice genes (LOC vs RAP) see ( http://rapdblegacy.dna.affrc.go.jp/gene/nomenclature.html); (^4^) Position of the candidate genes in barley according to the Barley Genome Zipper. (^5^) Position of the candidate genes in *H*. *chilense* map. Genes in bold are shown in the Barley Genome Zipper [56]; *nd*: not determined.

Physical mapping by using the series of addition lines of *H*. *chilense* in common wheat was also possible for genes *Hdr*, *Ggpps1*, *Psy2*, *Zds* and *Ccd1*. Physical location could not be confirmed for genes located on chromosomes 2H^ch^L and 3H^ch^ (since there are no addition lines for these chromosomes) or when polymorphism with wheat was not detected with the designed markers (*b*-*Lcy*, *Psy3*, *Pds* and *Ppo1*).

To study the macrocolinearity between *H*. *chilense* and barley for these candidate genes, the *Poaceae* orthologous group corresponding to each candidate gene was identified from http://rice.plantbiology.msu.edu/annotation_pseudo_pog.shtml using the rice gene as query (Table 3). Orthologous genes in *Brachypodium* and *Sorghum* were identified by this means. The RAP locus identifier was retrieved using the ID Converter tool ( http://rapdb.dna.affrc.go.jp/tools/converter). Rice, barley and sorghum orthologs were located in the Barley Genome Zipper [56], which allowed the determination of their relative positions in the barley map [57] in all cases except for *Zds* which was not found in the Barley Genome Zipper (Table 3). The *Psy1*, previously mapped in *H*. *chilense*, was also located in barley.

### Determination of YPC regions in *H*. *chilense*

The YPC content was determined in the H1×H7 RIL mapping population. Kruskall-Wallis (KW) association test revealed three main chromosome regions related to YPC (Figure 1). The largest is located on chromosome 2H^ch^ where the majority of markers showed an association with YPC (Figure 1). Smaller regions were detected on chromosomes 3H^ch^ and 7H^ch^. Candidate genes *Ggpps1*, *Zds*, *Hyd3*, *Ppo1*, *e*-*Lcy* and *Psy1* showed a significant association with YPC (Figure 1). The MQM analysis resulted in a significant QTL at the distal part of chromosome 2H^ch^ between 207 and 229 cM (*p*<0.05) where no carotenoid-related genes were mapped in this work.

## Discussion

### Macrocolinearity between *H*. *chilense* and *H*. *vulgare*

Comparative analysis of the twelve candidate genes positions in *H*. *chilense* and barley using the Barley Genome Zipper [56] revealed a good agreement between both species for the candidate genes selected (Table 3). Indeed, chromosome location and relative positions of genes within chromosomes were conserved in all cases. Regarding *Zds*, its position could not be established in barley since the orthologous genes from rice, sorghum or *Brachypodium* were not found in the Barley Genome Zipper [56]. However, the close linkage between *Zds* and *Ggpps1* in *H*. *chilense* suggests that the two genes are located in equivalent positions in barley. This is supported by the high-resolution comparative analysis between barley and rice performed by [56]. Briefly, the Barley Genome Zipper establishes the putative linear order of 21,766 barley genes by integrating gene indices of rice, sorghum and *Brachypodium*[56] and allows the establishment of the synteny relations between barley and rice chromosomes based on the position of the putative orthologs in both species. Chromosome 2H in barley corresponds to chromosome 4R in rice, except for a region in 2HS arm which is equivalent to 7R. In barley the region near the 2H centromere shows significant matches to both 7RS (where *Zds* is located in rice) and 7RL (where *Ggpps1* is located in rice). This is in agreement with *H*. *chilense* results and suggests that *Zds* would be located in an equivalent position in barley.

An apparent break in the synteny between *H*. *chilense* and *H*. *vulgare* happens for *Psy1* which is located in 7HL in barley and in 7H^ch^α in *H*. *chilense*[31]. However, genetic mapping could not establish the relative position of *Psy1* in 7H^ch^ chromosome since it remained in a short linkage group not linked to 7H^ch^ chromosome. Thus, its putative location in 7H^ch^α is based upon physical mapping using a telosomic addition line. We have recently observed a reorganization of the *H*. *chilense* chromatin in wheat background between different chromosomes (A. Martín, personal communication). Thus, a translocation involving *Psy1* and 7H^ch^S during the development of the addition line cannot be totally excluded and further work is required to confirm the position of *Psy1* in the chromosome 7H^ch^.

### YPC regions: comparative analysis between *H*. *chilense* and wheat

The association study for YPC allowed the identification of three main regions in chromosomes 2H^ch^, 3H^ch^ and 7H^ch^ for YPC variation in *H*. *chilense* as revealed by a KW association test. Despite a single QTL at the distal part of chromosome 2H^ch^L being above the significance threshold established, all three regions were considered significant since the population size critically affects the power of QTL detection and the precision of QTL estimates [58]. For instance, QTLs with small effects for stripe rust resistance in barley were detected only by increasing the population size [59]. The significance threshold must thus be chosen according to the population size and the trait of interest [58]. Therefore, we relied on KW-defined regions and considered the QTL analyses to conclude that the distal part of chromosome 2H^ch^ was the most significant one. Seven of the candidate genes were not associated with YPC in this work. This may indicate that they do not contribute to YPC variation in this population. Alternatively, these genes might have a minor effect which cannot be detected with this population size.

The region located in chromosome 3H^ch^ includes the *eLcy* gene, whose role in YPC has been demonstrated in hexaploid wheat and maize. In wheat, *eLcy* co-localized with a QTL for lutein accumulation and a specific mutation causing lutein content variations was identified [35]. This QTL was located in the proximal 3BS region this being the position of this gene in hexaploid wheat in agreement with the results obtained in *H*. *chilense*. Similarly, it has been shown that a QTL for carotenoid accumulation in maize chromosome 8 co-locates with *eLcy* gene [25,26].

The distal part of chromosome 7H^ch^L is also related to YPC in *H*. *chilense*. The importance of this region has been clearly demonstrated in wheat, as a result of variations in *Psy1* genes [32,33,38]. This has promoted extensive work towards the identification of *Psy1A* and *Psy1B* alleles, the determination of their role in YPC and the development of functional markers for selection [34,60,61]. Furthermore, there is evidence of an additional locus for YPC in the distal part of chromosome 7AL in durum wheat [38]. Given that *Psy1* is apparently located in the 7H^ch^α arm, this might indicate that an additional locus is found at 7H^ch^L, in agreement with durum wheat results. Nevertheless, since genetic mapping did not resolve *Psy1* position within chromosome 7H^ch^, we cannot totally discard the occurrence of a translocation 7H^ch^L/7H^ch^S during the development of the wheat/*H*. *chilense* chromosome addition line. Thus, macrocolinearity cannot be established for this gene at present and the YPC region on chromosome 7H^ch^L might possibly be related to *Psy1* variation. Indeed, *Psy1* showed a low association with YPC (*p*<0.1).

Finally, there is a region on chromosome 2H^ch^ related to YPC which extends over the 80% of the chromosomal length. Four candidate genes, *Ggpps1*, *Zds*, *Hyd3* and *Ppo1*, are mapped within this interval. The most significant zone within this region is located in the distal part of chromosome 2H^ch^L (Figure 1). However, the complete KW-defined region must be considered as a target since the population size is probably hampering the determination of other QTLs in this chromosome. Indeed, in a preliminary map constructed using a H1×H7 F_2_ population, which did not cover the distal part of chromosome 2H^ch^, a QTL for YPC was detected in the chromosome 2H^ch^ in the vicinity of the marker *IASpHc21*[62] which is now mapped at 48 cM (Figure 1). Additionally, the location of four carotenoid-related genes in this area further supports the importance of this region for YPC.

The relevance of chromosome 2A for YPC and/or carotenoid content has been previously reported in wheat. Most works have focused on the role of polyphenol oxidases since these enzymes cause the darkening of flour, pasta and noodle products [63]. Duplicated polyphenol oxidase genes (*Ppo1* and *Ppo2*) have been identified and mapped clustered in barley 2H chromosome [64]. Although only *Ppo1* was mapped in *H*. *chilense*, a tightly linked *Ppo2* may be expected. In any case, the association of *Ppo1* and YPC in *H*. *chilense* seems to indicate the occurrence of darkening processes in this species. Similarly, the role of *PpoA1* and *PpoD1* has been extensively studied in wheat including mapping studies [65,66], the development of functional markers [67-69] and the cloning and analysis of these genes [70,71]. Recently, duplicated *Ppo* genes (*PpoA2*, *PpoB2* and *PpoD2*) have been identified [72] and mapped to chromosomes 2A, 2B and 2D [73]. Thus the results obtained in *H*. *chilense* are in agreement with previous findings in wheat.

Our results also suggest the potential importance of the centromeric region on chromosome 2H^ch^ for YPC. QTL and association mapping studies have identified regions for YPC or carotenoid content in chromosome 2A [32,33,74]. Recently, a QTL on the centromeric region of chromosome 2A has been reported [33]. However, it could not be associated with YPC *per se* since the markers within the confidence interval were related to kernel weight variation. Thus, it was proposed that this QTL may be associated with yellow pigment concentration due to a lower carbohydrate content in smaller grains or it could be linked to a QTL for kernel weight [33]. Since there is no significant association between YPC and kernel weight in *H*. *chilense*[42], this region does not seem to be related to carotenoid concentration. Moreover, previous findings in durum wheat also support the existence of a QTL for YPC in this region [32,74]. Both studies showed the association between *Xgwm495* and YPC using a mapping population derived from the cross W9262-260D3 × Kofa [32] and a collection of 93 diverse accessions of durum wheat [74]. This marker is located in the arm 2AS (C-2AS5-078 bin) at 108.5 cM [75] (see Group 2 data file at http://wheat.pw.usda.gov/ggpages/SSRclub/GeneticPhysical/) between markers *Xgwm372* (C-2AS5-078 bin, at 90.8 cM) and *Xgwm249* (C-2AL-1-0.85 bin, at 111.9 cM). Both *Xgwm372* and *Xgwm249* are within the confidence interval for the QTL on chromosome 2A reported by [33]. Hence, all three works identify the same region around the centromere of chromosome 2A, and thus, this region seems to be effectively related to YPC and/or carotenoid content in durum wheat. The results presented in this work also support the importance of the centromeric region of the chromosome 2H^ch^ in YPC. Furthermore, the *Zds* and *Ggpps1* genes were mapped within this region, and they should therefore be considered in durum wheat. The potential association of *Zds* with YPC in durum wheat has already been hypothesized after physical mapping on chromosomes 2A and 2B [76]. Later studies cloned *Zds* gene in common wheat [77] and identified a QTL for YPC related to *ZdsD1* variation in chromosome 2D in common wheat [78]. Similarly, the co-localization of *Zds* with a QTL for carotenoid content in chromosome 7 of maize [25] also supports the potential role of this gene in grasses. Similarly, the association between *Ggpps1* expression and the carotenoid content in maize [29] and the close linkage between *Zds* and *Ggpps1* in *H*. *chilense* make the latter an interesting candidate gene for durum wheat studies. Likewise, variations at *Hyd3* locus are important for maize endosperm carotenoid content [28,79]. Given the association of this gene with YPC and its position in *H*. *chilense* map, *Hyd3* should be also considered in further studies in durum wheat.

The most significant region of 2H^ch^, as identified by MQM analysis, is located in the distal part of the chromosome 2H^ch^L where no candidate genes were located in this work. The occurrence of this association can be explained by several hypotheses, although they are all very speculative. The simplest one is that other carotenoid genes not considered in this study are located in the distal 2H^ch^L. A second possibility would be that genes for carotenoid esterification are located in this region. A QTL for lutein esters was identified in chromosome 2B [35]. It is flanked by the marker KsuD22 which has been located in the chromosome 2BL by deletion mapping [75]. Esterification is a common mechanism for sequestering carotenoids in plants [80]. Indeed, carotenoids are accumulated in specialized lipoprotein-sequestering structures in the chromoplasts [81] and the apolar compounds of some chromoplasts, such as fibrils, are mainly composed of esterified xanthophylls [81]. *H*. *chilense* genome addition in tritordeums produces both high carotenoid content and an increase in lutein esterification [10], and, thus, the presence of genes for differential esterification ability in the 2H^ch^L chromosome might be considered in the future.

The lack of association between the rest of the candidate genes (*Dxr*, *Hdr*, *Ccd1*, *bLcy*, *Pds*, *Psy2* and *Psy3*) and YPC in *H*. *chilense* suggests at least two possibilities: (1) these genes are not related with YPC variation in the population; (2) they have minor effects which cannot be detected with the current population size. With the results presented in this work it is not possible to discern between these possibilities. While *Psy2* and *Psy3* are not associated with seed carotenoid content in other grasses (reviewed by [82]) and thus, they may be discarded for further analyses, the rest of the genes might be still useful for durum wheat studies. For instance, *Ccd1* copy number is negatively correlated with carotenoid content in maize [83]. It is mapped in the distal part of 5H^ch^S in concordance with QTLs for carotenoid content and YPC in durum wheat [33,84]. Thus, it may be an interesting candidate gene for durum wheat despite the lack of association with YPC in this work.

## Conclusions

The equivalent location of the carotenoid-related genes in *H*. *chilense* and barley shows a high level of collinearity between both species, at least for the genes studied in this work. Considering the high synteny among *Triticeae* species along with the high YPC content of *H*. *chilense* and its derived amphiploids (tritordeums), the H^ch^ genome constitutes a valuable tool for YPC and carotenoid content studies in the *Triticeae*. Indeed, the YPC regions detected in *H*. *chilense* showed good agreement with previous findings in wheat. Accordingly, the location of *Zds* and *Ggpps1* within one of these regions around the 2H^ch^ centromere is in concordance with QTL for YPC and carotenoid content in wheat, and suggests that both genes should be considered for durum wheat improvement. In addition, the identification and mapping of the candidate genes in *H*. *chilense* will enable further studies to investigate the genetic basis of the high YPC/carotenoid content in tritordeum which could also be extrapolated to durum wheat.

## Abbreviations

*Dxr*: 1-deoxy-D-xylulose 5-phosphate reductoisomerase; *Hdr* synonym *ispH*: 4-hydroxy-3-methylbut-2-enyl diphosphate reductase; ACS: Automatic cofactor selection; *Hyd3*: *Beta*-carotene hydroxylase 3; *Ccd1*: Cleavage dioxygenase 1 gene; CAP: Cleaved Amplified Polymorphism; DArT: Diversity Arrays Technology; *Ggpps1*: Geranyl geranyl pyrophosphate synthase; IM: Interval Mapping; KW: Kruskall-Wallis; *b*-*Lcy*: Lycopene *beta* cyclase; *e*-*Lcy*: Lycopene epsilon cyclase; MEP: Methylerythritol phosphate; ML-mapping: Monte Carlo Maximum Likelihood Mapping algorithm; MQM: Multiple QTL Mapping; *Pds*: Phytoene dehydrogenase; *Psy2*: Phytoene synthase 2; *Psy3*: Phytoene synthase 3; *Ppo1*: Polyphenol oxidase 1 gene; QTL: Quantitative trait locus; RILs: Recombinant Inbred Lines; SNP: Single Nucleotide Polymorphisms; YPC: Yellow pigment content; *Zds*: *Zeta*-carotene desaturase.

## Competing interests

The authors declare that they have no competing interests.

## Authors’ contributions

CR-S and SGA conceived and designed the study. CR-S performed the experiments. SGA performed the QTL analyses. CR-S and SGA analyzed the data and wrote the paper. Both authors have read and approved the final manuscript.

## Supplementary Material

Additional file 1 **Table S1.** Accession numbers of the sequences used for alignments in the design of primers for amplification of the orthologous genes in *H*. *chilense.* Click here for file

## References

[B1] Martin AC Atienza SG Ramirez MC Barro F Martin A Male fertility restoration of wheat in *Hordeum chilense* cytoplasm is associated with 6H(ch)S chromosome addition Aust J Agric Res 2008 59 206 213 10.1071/AR07239

[B2] Martin AC Atienza SG Ramirez MC Barro F Martin A Chromosome engineering in wheat to restore male fertility in the msH1 CMS system Mol Breed 2009 24 397 408 10.1007/s11032-009-9301-z

[B3] Martín AC Atienza SG Ramírez M Barro F Martin A Molecular and cytological characterization of an extra acrocentric chromosome that restores male fertility of wheat in the msH1 CMS system Theor Appl Genet 2010 121 1093 1101 10.1007/s00122-010-1374-x 20549484

[B4] Alvarez JB Martin LM Martin A Chromosomal localization of genes for carotenoid pigments using addition lines of *Hordeum chilense* in wheat Plant Breed 1998 117 287 289 10.1111/j.1439-0523.1998.tb01942.x

[B5] Martin A Martínez C Rubiales D Ballesteros J Güedes-Pinto H, Darvey N, Carnide VP *Tritordeum: triticale's* new brother cereal Triticale: today and tomorrow 1996 Dordrecht, NL: Kluwer Academic Publishers 57 72

[B6] Rodríguez-Suárez C Giménez MJ Ramírez MC Martín AC Gutierrez N Ávila CM Martín A Atienza SG Exploitation of nuclear and cytoplasm variability in *Hordeum chilense* for wheat breeding Plant Genet Res Charact Utiliz 2011 9 313 316

[B7] Martin A Chapman V A hybrid between *Hordeum chilense* and *Triticum aestivum* Cereal Res Commun 1977 5 365 368

[B8] Martin A Sánchez-Monge Laguna E Cytology and morphology of the amphiploid *Hordeum chilense* × *Triticum turgidum* conv *durum* Euphytica 1982 31 261 267 10.1007/BF00028329

[B9] Martin A Alvarez JB Martin LM Barro F Ballesteros J The development of tritordeum: a novel cereal for food processing J Cereal Sci 1999 30 85 95 10.1006/jcrs.1998.0235

[B10] Atienza SG Ballesteros J Martin A Hornero-Mendez D Genetic variability of carotenoid concentration and degree of esterification among tritordeum (x*Tritordeum* Ascherson et Graebner) and durum wheat accessions J Agric Food Chem 2007 55 4244 4251 10.1021/jf070342p 17439153

[B11] Digesù AM Platani C Cattivelli L Mangini G Blanco A Genetic variability in yellow pigment components in cultivated and wild tetraploid wheats J Cereal Sci 2009 50 210 218 10.1016/j.jcs.2009.05.002

[B12] Hentschel V Kranl K Hollmann J Lindhauer MG Bohm V Bitsch R Spectrophotometric determination of yellow pigment content and evaluation of carotenoids by high-performance liquid chromatography in durum wheat grain J Agric Food Chem 2002 50 6663 6668 10.1021/jf025701p 12405758

[B13] Leenhardt F Lyan B Rock E Boussard A Potus J Chanliaud E Remesy C Genetic variability of carotenoid concentration, and lipoxygenase and peroxidase activities among cultivated wheat species and bread wheat varieties Eur J Biochem 2006 25 170 176

[B14] Al-Delaimy WK Slimani N Ferrari P Key T Spencer E Johansson I Johansson G Mattisson I Wirfalt E Sieri S Plasma carotenoids as biomarkers of intake of fruits and vegetables: ecological-level correlations in the European Prospective Investigation into Cancer and Nutrition (EPIC) Eur J Clin Nutr 2005 59 1397 1408 10.1038/sj.ejcn.1602253 16160701

[B15] Nishino H Murakoshi M Tokuda H Satomi Y Cancer prevention by carotenoids Arch Biochem Biophys 2009 483 165 168 10.1016/j.abb.2008.09.011 18848517

[B16] Giuliano G Tavazza R Diretto G Beyer P Taylor MA Metabolic engineering of carotenoid biosynthesis in plants Trends Biotechnol 2008 26 139 145 10.1016/j.tibtech.2007.12.003 18222560

[B17] Carpentier S Knaus M Suh M Associations between lutein, zeaxanthin, and age-related macular degeneration: An overview Crit Rev Food Sci Nutr 2009 49 313 326 10.1080/10408390802066979 19234943

[B18] Fraser PD Bramley PM The biosynthesis and nutritional uses of carotenoids Prog Lipid Res 2004 43 228 265 10.1016/j.plipres.2003.10.002 15003396

[B19] Cuttriss A Mimica J Howitt C Pogson B Wise RR, Hoober JK, Dordrecht Carotenoids The structure and function of plastids 2006 The Netherlands: Springer 315 334

[B20] Hirschberg J Carotenoid biosynthesis in flowering plants Curr Opin Plant Biol 2001 4 210 218 10.1016/S1369-5266(00)00163-1 11312131

[B21] Howitt CA Pogson BJ Carotenoid accumulation and function in seeds and non-green tissues Plant Cell Environ 2006 29 435 445 10.1111/j.1365-3040.2005.01492.x 17080597

[B22] Matthews PD Wurtzel ET Socaciu C Biotechnology of food colorant production Food Colorants: Chemical and Functional Properties 2007 Boca Raton, FL: CRC Press 347 398

[B23] Buckner B San Miguel P Janick-Buckner D Bennetzen JL The y1 gene of maize codes for phytoene synthase Genetics 1996 143 479 488 8722797 10.1093/genetics/143.1.479 PMC1207279

[B24] Gallagher CE Matthews PD Li F Wurtzel ET Gene duplication in the carotenoid biosynthetic pathway preceded evolution of the grasses Plant Physiol 2004 135 1776 1783 10.1104/pp.104.039818 15247400 PMC519089

[B25] Wong JC Lambert RJ Wurtzel ET Rocheford TR QTL and candidate genes phytoene synthase and zeta-carotene desaturase associated with the accumulation of carotenoids in maize Theor Appl Genet 2004 108 349 359 10.1007/s00122-003-1436-4 14523521

[B26] Harjes CE Rocheford TR Bai L Brutnell TP Kandianis CB Sowinski SG Stapleton AE Vallabhaneni R Williams M Wurtzel ET Natural genetic variation in lycopene epsilon cyclase tapped for maize biofortification Science 2008 319 330 333 10.1126/science.1150255 18202289 PMC2933658

[B27] Li F Murillo C Wurtzel ET Maize Y9 encodes a product essential for 15-cis-{zeta}-carotene isomerization Plant Physiol 2007 144 1181 1189 10.1104/pp.107.098996 17434985 PMC1914175

[B28] Vallabhaneni R Gallagher CE Licciardello N Cuttriss AJ Quinlan RF Wurtzel ET Metabolite sorting of a germplasm collection reveals the hydroxylase3 locus as a new target for maize provitamin A biofortification Plant Physiol 2009 151 1635 1645 10.1104/pp.109.145177 19767386 PMC2773064

[B29] Vallabhaneni R Wurtzel ET Timing and biosynthetic potential for carotenoid accumulation in genetically diverse germplasm of maize Plant Physiol 2009 150 562 572 10.1104/pp.109.137042 19346441 PMC2689957

[B30] Fraser PD Truesdale MR Bird CR Schuch W Bramley PM Carotenoid biosynthesis during tomato fruit development (evidence for tissue-specific gene expression) Plant Physiol 1994 105 405 413 12232210 10.1104/pp.105.1.405 PMC159369

[B31] Atienza SG Avila CM Martin A The development of a PCR-based marker for *Psy1* from *Hordeum chilense*, a candidate gene for carotenoid content accumulation in tritordeum seeds Austr J Agric Res 2007 58 767 773 10.1071/AR06338

[B32] Pozniak CJ Knox RE Clarke FR Clarke JM Identification of QTL and association of a phytoene synthase gene with endosperm colour in durum wheat Theor Appl Genet 2007 114 525 537 10.1007/s00122-006-0453-5 17131106

[B33] Blanco A Colasuonno P Gadaleta A Mangini G Schiavulli A Simeone R Digesù AM De Vita P Mastrangelo AM Cattivelli L Quantitative trait loci for yellow pigment concentration and individual carotenoid compounds in durum wheat J Cereal Sci 2011 54 255 264 10.1016/j.jcs.2011.07.002

[B34] He XY Zhang YL He ZH Wu YP Xiao YG Ma CX Xia XC Characterization of phytoene synthase 1 gene (*Psy1*) located on common wheat chromosome 7A and development of a functional marker Theor Appl Genet 2008 116 213 221 10.1007/s00122-007-0660-8 17943267

[B35] Howitt CA Cavanagh CR Bowerman AF Cazzonelli C Rampling L Mimica JL Pogson BJ Alternative splicing, activation of cryptic exons and amino acid substitutions in carotenoid biosynthetic genes are associated with lutein accumulation in wheat endosperm Func Integr Genomics 2009 9 363 376 10.1007/s10142-009-0121-3 19330366

[B36] Singh A Reimer S Pozniak CJ Clarke FR Clarke JM Knox RE Singh AK Allelic variation at *Psy1-A1* and association with yellow pigment in durum wheat grain Theor Appl Genet 2009 118 1539 1548 10.1007/s00122-009-1001-x 19319504

[B37] Zhang W Chao S Manthey F Chicaiza O Brevis JC Echenique V Dubcovsky J QTL analysis of pasta quality using a composite microsatellite and SNP map of durum wheat Theor Appl Genet 2008 117 1361 1377 10.1007/s00122-008-0869-1 18781292

[B38] Zhang W Dubcovsky J Association between allelic variation at the Phytoene synthase 1 gene and yellow pigment content in the wheat grain Theor Appl Genet 2008 116 635 645 10.1007/s00122-007-0697-8 18193186

[B39] Zhang YL Wu YP Xiao YG He ZH Zhang Y Yan J Xia XC Ma CX QTL mapping for flour and noodle colour components and yellow pigment content in common wheat Euphytica 2009 165 435 444 10.1007/s10681-008-9744-z

[B40] Bolot S Abrouk M Masood-Quraishi U Stein N Messing J Feuillet C Salse J The ‘inner circle’ of the cereal genomes Curr Opin Plant biol 2009 12 119 125 10.1016/j.pbi.2008.10.011 19095493

[B41] Devos KM Updating the 'Crop Circle' Curr Opin Plant Biol 2005 8 155 162 10.1016/j.pbi.2005.01.005 15752995

[B42] Alvarez JB Martin LM Martin A Genetic variation for carotenoid pigment content in the amphiploid *Hordeum chilense* x *Triticum turgidum* conv. *durum* Plant Breed 1999 118 187 189 10.1046/j.1439-0523.1999.118002187.x

[B43] Atienza SG Avila CM Ramirez MC Martin A Application of near infrared reflectance spectroscopy to the determination of carotenoid content in tritordeum for breeding purposes Austr J Agric Res 2005 56 85 89 10.1071/AR04154

[B44] Pudoc Broertjes C Miller TE Reader SM Chapman V The addition of *Hordeum chilense* chromosomes to wheat Induced variability in plant breeding 1982 Wageningen, The Netherlands: Proceedings of the International Eucarpia Symposium 79 81

[B45] Cherif-Mouaki S Said M Alvarez JB Cabrera A Sub-arm location of prolamin and EST-SSR loci on chromosome 1H^ch^ from *Hordeum chilense* Euphytica 2011 178 63 69 10.1007/s10681-010-0268-y

[B46] Said M Cabrera A A physical map of chromosome 4H(ch) from *H. chilense* containing SSR, STS and EST-SSR molecular markers Euphytica 2009 167 253 259 10.1007/s10681-009-9895-6

[B47] Rodríguez-Suárez C Giménez MJ Gutiérrez N Ávila CM Machado A Huttner E Ramírez MC Martín A Castillo A Kilian A Development of wild barley (*Hordeum chilense*)-derived DArT markers and their use into genetic and physical mapping Theor Appl Genet 2012 124 713 722 10.1007/s00122-011-1741-2 22048641

[B48] Murray YHG Thompson WF Rapid isolation of high molecular weight plant DNA Nucleic Acids Res 1980 8 4321 4326 10.1093/nar/8.19.4321 7433111 PMC324241

[B49] Rodríguez-Suárez C Atienza SG Pistón F Allelic variation, alternative splicing and expression analysis of *Psy1* gene in *Hordeum chilense* Roem. et Schult PLoS One 2011 6 e19885 10.1371/journal.pone.0019885 21603624 PMC3095628

[B50] Untergasser A Nijveen H Rao X Bisseling T Geurts R Leunissen JAM Primer3Plus, an enhanced web interface to Primer3 Nucleic Acids Res 2007 35 W71 W74 10.1093/nar/gkm306 17485472 PMC1933133

[B51] AACC AACC Method 14–50 1997

[B52] Van Ooijen JW Kiazma BW Joinmap 4.0®, Software for the calculation of genetic linkage maps in experimental populations 2006 Wageningen The Netherlands: Plant Research International

[B53] Van Ooijen JW Boer MP Jansen RC Maliepaard C Kiazma BW MapQTLTM version 4.0: Software for the calculation of QTL positions on genetic maps 2000 Wageningen, The Netherlands: Plant Research International

[B54] Van Ooijen JW Accuracy of mapping quantitative trait loci in autogamous species Theor Appl Genet 1992 84 803 811 10.1007/BF00227388 24201478

[B55] Voorrips RE MapChart: Software for the graphical presentation of linkage maps and QTLs J Hered 2002 93 77 78 10.1093/jhered/93.1.77 12011185

[B56] Mayer KFX Martis M Hedley PE Šimková H Liu H Morris JA Steuernagel B Taudien S Roessner S Gundlach H Unlocking the barley genome by chromosomal and comparative genomics Plant Cell 2011 23 1249 1263 10.1105/tpc.110.082537 21467582 PMC3101540

[B57] Close T Bhat P Lonardi S Wu Y Rostoks N Ramsay L Druka A Stein N Svensson J Wanamaker S Development and implementation of high-throughput SNP genotyping in barley BMC Genomics 2009 10 582 10.1186/1471-2164-10-582 19961604 PMC2797026

[B58] Schön CC Utz HF Groh S Truberg B Openshaw S Melchinger AE Quantitative trait locus mapping based on resampling in a vast maize testcross experiment and its relevance to quantitative genetics or complex traits Genetics 2004 167 485 498 10.1534/genetics.167.1.485 15166171 PMC1470842

[B59] Vales MI Schön CC Capettini F Chen XM Corey AE Mather DE Mundt CC Richardson KL Sandoval-Islas JS Utz HF Effect of pupulation size on the estimation of QTL: a test using resistance to barley stripe rust Theor Appl Genet 2005 111 1260 1270 10.1007/s00122-005-0043-y 16179997

[B60] He XY He ZH Ma W Appels R Xia XC Allelic variants of phytoene synthase 1 (*Psy1*) genes in Chinese and CIMMYT wheat cultivars and development of functional markers for flour colour Mol Breed 2009 23 553 563 10.1007/s11032-009-9255-1

[B61] He XY Wang JW Ammar K Pena RJ Xia XC He ZH Allelic variants at the *Psy-A1* and *Psy-B1* loci in durum wheat and their associations with grain yellowness Crop Sci 2009 49 2058 2064 10.2135/cropsci2008.11.0651

[B62] Atienza SG Ramirez CM Hernandez P Martin A Chromosomal location of genes for carotenoid pigments in Hordeum chilense Plant Breed 2004 123 303 304 10.1111/j.1439-0523.2004.00918.x

[B63] McIntosh RA Yamazaki Y Dubcovsky J Rogers J Morris C Somers DJ Appels R Devos KM Catalogue of gene symbols for wheat http://wheat.pw.usda.gov/GG2/Triticum/wgc/2008/

[B64] Taketa S Matsuki K Amano S Saisho D Himi E Shitsukawa N Yuo T Noda K Takeda K Duplicate polyphenol oxidase genes on barley chromosome 2H and their functional differentiation in the phenol reaction of spikes and grains J Exp Bot 2010 61 3983 3993 10.1093/jxb/erq211 20616156 PMC2935872

[B65] Jimenez M Dubcovsky J Chromosome location of genes affecting polyphenol oxidase activity in seeds of common and durum wheat Plant Breed 1999 118 395 398 10.1046/j.1439-0523.1999.00393.x

[B66] Mares DJ Campbell AW Mapping components of flour colour in Australian wheat Austr J Agric Res 2001 52 1297 1309 10.1071/AR01048

[B67] He XY He ZH Zhang LP Sun DJ Morris CF Fuerst EP Xia XC Allelic variation of polyphenol oxidase (PPO) genes located on chromosomes 2A and 2D and development of functional markers for the PPO genes in common wheat Theor Appl Genet 2007 115 47 58 10.1007/s00122-007-0539-8 17426955

[B68] Sun D He Z Xia X Zhang L Morris C Appels R Ma W Wang H A novel STS marker for polyphenol oxidase activity in bread wheat Mol Breed 2005 16 209 218 10.1007/s11032-005-6618-0

[B69] Chang C Zhang H-P Xu J You M-S Li B-Y Liu G-T Variation in two PPO genes associated with polyphenol oxidase activity in seeds of common wheat Euphytica 2007 154 181 193 10.1007/s10681-006-9285-2

[B70] He XY He ZH Morris CF Xia XC Cloning and phylogenetic analysis of polyphenol oxidase genes in common wheat and related species Genet Res Crop Evol 2009 56 311 321 10.1007/s10722-008-9365-3

[B71] Sun YW He ZH Ma WJ Xia XC Alternative splicing in the coding region of Ppo-A1 directly influences the polyphenol oxidase activity in common wheat (*Triticum aestivum* L.) Funct Integr Genomics 2011 11 85 93 10.1007/s10142-010-0201-4 21046181

[B72] Beecher B Skinner DZ Molecular cloning and expression analysis of multiple polyphenol oxidase genes in developing wheat (*Triticum aestivum*) kernels J Cereal Sci 2011 53 371 378 10.1016/j.jcs.2011.01.015

[B73] Beecher B Carter A See D Genetic mapping of new seed-expressed polyphenol oxidase genes in wheat (*Triticum aestivum* L.) Theor Appl Genet 2012 124 1463 1473 10.1007/s00122-012-1801-2 22311372

[B74] Reimer S Pozniak CJ Clarke FR Clarke JM Somers DJ Knox RE Singh AK Association mapping of yellow pigment in an elite collection of durum wheat cultivars and breeding lines Genome 2008 51 1016 1025 10.1139/G08-083 19088814

[B75] Sourdille P Singh S Cadalen T Brown-Guedira GL Gay G Qi L Gill BS Dufour P Murigneux A Bernard M Microsatellite-based deletion bin system for the establishment of genetic-physical map relationships in wheat (*Triticum aestivum* L.) Func Integr Genomics 2004 4 12 25 10.1007/s10142-004-0106-1 15004738

[B76] Cenci A Somma S Chantret N Dubcovsky J Blanco A PCR identification of durum wheat BAC clones containing genes coding for carotenoid biosynthesis enzymes and their chromosome localization Genome 2004 47 911 917 10.1139/g04-033 15499405

[B77] Cong L Wang C Chen L Liu H Yang G He G Expression of phytoene synthase1 and carotene desaturase crtI genes result in an increase in the total carotenoids content in transgenic elite wheat (*Triticum aestivum* L.) J Agric Food Chem 2009 57 8652 8660 10.1021/jf9012218 19694433

[B78] Zhang CY Dong CH He XY Zhang LP Xia XC He ZH Allelic variants at the *TaZds-D1* locus on wheat chromosome 2DL and their association with yellow pigment content Crop Sci 2011 51 1580 1590 10.2135/cropsci2010.12.0689

[B79] Yan JB Kandianis CB Harjes CE Bai L Kim EH Yang XH Skinner DJ Fu ZY Mitchell S Li Q Rare genetic variation at Zea mays *crtRB1* increases beta-carotene in maize grain Nature Genet 2010 42 322 U374 10.1038/ng.551 20305664

[B80] Hornero-Mendez D Minguez-Mosquera MI Xanthophyll esterification accompanying carotenoid overaccumulation in chromoplast of *Capsicum annuum* ripening fruits is a constitutive process and useful for ripeness index J Agric Food Chem 2000 48 1617 1622 10.1021/jf9912046 10820068

[B81] Vishnevetsky M Ovadis M Vainstein A Carotenoid sequestration in plants: the role of carotenoid-associated proteins Trends Plant Sci 1999 4 232 235 10.1016/S1360-1385(99)01414-4 10366880

[B82] Rodríguez-Suárez C Giménez MJ Atienza SG Progress and perspectives for carotenoid accumulation in selected Triticeae species Crop Pasture Sci 2010 61 743 751 10.1071/CP10025

[B83] Vallabhaneni R Bradbury LMT Wurtzel ET The carotenoid dioxygenase gene family in maize, sorghum, and rice Arch Biochem Biophys 2010 504 104 111 10.1016/j.abb.2010.07.019 20670614 PMC2957549

[B84] Roncallo P Cervigni GL Jensen C Miranda R Carrera AD Helguera M Echenique V QTL analysis of main and epistatic effects for flour color traits in durum wheat Euphytica 2012 185 1 77 92 10.1007/s10681-012-0628-x

