# Supplementary material for: Hordeum chilense genome, a useful tool to investigate the endosperm yellow pigment content in the Triticeae
Source: BMC Plant Biol. 2012 Nov 2;12:200. doi: 10.1186/1471-2229-12-200 (PMC3534404; doi:10.1186/1471-2229-12-200)
Supplement: Additional file 1 — Table S1. Accession numbers of the sequences used for alignments in the design of primers for amplification of the orthologous genes in H. chilense. [file 1471-2229-12-200-S1.docx]

**Table S1** Accession numbers of the main sequences used for alignments in the design of primers for amplification of the corresponding orthologous genes in *H. chilense*

| **Genes** | **Rice** | **Maize** | **Sorghum** | **Wheat** | **Barley** |
| --- | --- | --- | --- | --- | --- |
| ***Dxr*** | NM_001048315 | AJ297566 |  | AK334065 | AJ583446 |
| ***Hdr*** | NM_001057702 | NM_001175829 | XM_002463888 |  | AK354331 |
| ***Ggpps1*** | NM_001066637 | NM_001197001 | XM_002463039 | AK334372 | AK363280 |
| ***Pds*** | NM_001055721 | L39266 |  | FJ517553 | AK371906 |
| ***Zds*** | NM_001065680 | AF047490 | AY714266 | FJ169496 |  |
| ***Psy2*** | NM_001073857 | NM_001114645 | XM_002442533 | DQ642441 | HM539709 |
| ***Psy3*** | NM_001070427 | NM_001114653 | AY705390 |  | AK366080 |
| ***e-Lcy*** | NM_001049945 | EU924262 | XM_002455793 | EU649785 | AK371513 |
| ***b-Lcy*** | NM_001052686 | AY206862 | XM_002453403 | AK334392 | AK373933 |
| ***Hyd3*** | NM_001060175 | AY844957 |  | AK332680 | AK366573 |
| ***Ccd1*** | NM_001073927 | DQ100347 |  |  | AK248749 |
| ***Ppo1*** |  |  |  | EF070147-50 | AB549330 |
